# Quantitative cardiac magnetic resonance standardized signal intensity comparison in dilated cardiomyopathy vs. cardiac sarcoidosis

**DOI:** 10.1007/s10840-025-02042-7

**Published:** 2025-05-06

**Authors:** Ting-Wei Ernie Liao, Lingyu Xu, Mirmilad Pourmousavi Khoshknab, Paul J. Mather, Paco E. Bravo, Benoit Desjardins, Saman Nazarian

**Affiliations:** 1 Section of Cardiac Electrophysiology, Division of Cardiovascular Medicine, Department of Medicine, University of Pennsylvania School of Medicine, 3400 Civic Center Boulevard, Philadelphia, PA 19104 USA; 2 Section of Cardiomyopathy, Division of Cardiovascular Medicine, Department of Medicine , University of Pennsylvania School of Medicine, Philadelphia, PA USA; 3 Section of Nuclear Cardiology and Cardiovascular Molecular Imaging, Division of Cardiovascular Medicine, Department of Medicine, University of Pennsylvania School of Medicine, Philadelphia, PA USA; 4 Department of Radiology, Centre Hospitalier de l’Université de Montréal, Montréal, Québec, Canada; 5 Section of Cardiac Electrophysiology, University of Pennsylvania Perelman School of Medicine, Hospital of the University of Pennsylvania Pavilion, Second Floor City Side, Office 6, One Convention Avenue, Philadelphia, PA 19104 USA

**Keywords:** Dilated cardiomyopathy, Cardiac sarcoidosis, Cardiac magnetic resonance, Non-ischemic cardiomyopathy

## Abstract

**Background:**

Dilated cardiomyopathy (DCM) and cardiac sarcoidosis (CS) manifest unique late gadolinium enhancement (LGE) patterns on cardiac magnetic resonance (CMR), indicative of different myocardial scar distributions. However, the overlap in these patterns due to their lack of specificity complicates differentiation. This study introduces a novel quantitative method employing *z*-score analysis of LGE-CMR intensity to objectively compare the spatial distribution of LGE intensity between DCM and CS.

**Methods:**

This retrospective study included 22 NICM patients (13 DCM, 9 CS) who underwent CMR before electrophysiology study from November 2018 to May 2023. LGE images were delineated into sub-endocardial, mid-myocardial, and sub-epicardial layers across anterior, lateral, inferior, and septal walls using the AHA 17-segment model. CMR signal intensities were standardized to *z*-scores (*z* = (*x* − *μ*)/*σ*), with *x* as the signal intensity for a specific myocardial segment, and *μ* and *σ* as the mean and SD for all LV myocardial segments, to map regional intensity variations.

**Results:**

Compared to DCM, CS patients exhibited significantly higher CMR signal intensity *z*-scores in the septum (*β* = 0.32, *p* = 0.009), particularly in the endocardial third of the right ventricular (RV) side (*β* = 0.56, *p* = 0.001). A *z*-score greater than 0.40 in this area was associated with a CS diagnosis, with an area under the ROC curve of 0.692 in fivefold cross-validation.

**Conclusion:**

Patients with CS exhibit higher affinity for contrast in the septum, particularly on the RV endocardium. Standardized analysis of CMR signal intensities provides a novel, quantitative method for distinguishing CS from DCM, with the former exhibiting higher CMR signal intensity *z*-scores in the septum.

**Graphical Abstract:**

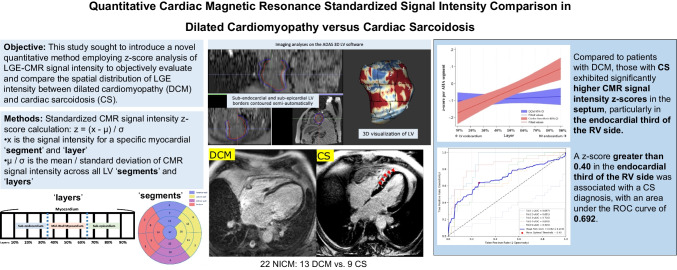

## Introduction

Non-ischemic cardiomyopathy (NICM) encompasses a diverse array of conditions resulting in myocardial dysfunction [1]. Precise identification of the specific underlying pathology is crucial for tailoring optimal patient care and predicting outcomes. Myocardial biopsy is limited by its invasiveness and poor diagnostic yield. The pattern of late gadolinium enhancement (LGE) on cardiovascular magnetic resonance (CMR) can help identify underlying conditions and is associated with all-cause and cardiovascular mortality, heart failure events, sudden cardiac death, and arrhythmic events among patients with NICM [2–6]. Consequently, current guidelines recommend the use of CMR imaging for the diagnosis of NICM, emphasizing the value of LGE in characterizing scar patterns to guide clinical decision-making and therapeutic interventions [1, 3].

Cardiac sarcoidosis and idiopathic dilated cardiomyopathy (DCM), two distinct etiological subtypes of NICM, are characterized by different patterns of LGE distribution [1, 3, 5, 7, 8]. In cardiac sarcoidosis, LGE patterns typically manifest as sub-epicardial and mid-wall involvement of the basal segments of the left ventricle (LV), exhibiting a patchy distribution; these patterns may also range from sub-endocardial to transmural involvement and can potentially extend to the inferolateral region of the LV or the right ventricular (RV) free wall [1, 7, 9]. In contrast, LGE associated with DCM is predominantly observed as a linear pattern within the mid-wall of the interventricular septum or as sub-epicardial involvement, although LV free wall involvement has also been noted [1, 3].

Despite its diagnostic utility, the interpretation of LGE in NICM is not without limitations, primarily due to the subjective nature of visual assessments of LGE and the challenges associated with direct quantitative comparisons of CMR signal intensities across different imaging platforms, stemming from variability in machine settings, contrast dose, renal function, hematocrit, and tissue properties [9, 10]. To address these challenges, our study introduces standardization of CMR LGE signal for quantitative analysis and comparison among NICM patients. This approach, previously utilized in other disease contexts but not in NICM, normalizes CMR voxel intensities by calculating the variation of the local mean signal intensity relative to the standard deviation [11, 12]. Our approach aims to provide a novel perspective on the characterization of myocardial substrate in NICM, extending beyond the conventional paradigm of LGE analysis.

## Methods

This study retrospectively identified patients with NICM due to DCM or cardiac sarcoidosis that had undergone CMR imaging between November 2018 and May 2023 prior to electrophysiology study for VT management or for risk stratification at the Hospital of the University of Pennsylvania. All demographic, procedural and follow up data prospectively entered a registry approved by the University of Pennsylvania’s Investigational Review Board for research use. The research reported in this article adhered to Helsinki Declaration guidelines.

### Diagnostic criteria of DCM and cardiac sarcoidosis

All patients were diagnosed according to the criteria established by the World Health Organization/International Society and Federation of Cardiology Task Force on the Definition and Classification of Cardiomyopathies [1, 13, 14]. Specifically, DCM was diagnosed based on echocardiographic evidence of LV enlargement, with LV end-diastolic dimensions exceeding the gender- and height-adjusted 95 th percentile, accompanied by a reduced LV ejection fraction (LVEF) of less than 50%. Patients with hypertrophic cardiomyopathy, restrictive cardiomyopathy, arrhythmogenic RV cardiomyopathy/dysplasia, or LV noncompaction were excluded. Testing for LMNA was restricted to those with inherited patterns of cardiomyopathy or concurrent neuromuscular and/or conduction disease. Patients included in the cardiac sarcoidosis group were required to meet the criteria for cardiac involvement and diagnostic guidelines for cardiac sarcoidosis outlined in the 2016 Japanese Circulation Society’s (JCS) guideline for the diagnosis and treatment of cardiac sarcoidosis [8]. Confirmation of cardiac involvement in JCS guideline was established through clinical findings categorized into major and minor criteria [8]. Patients with coronary artery disease or cardiomyopathies due to other causes, such as valvular heart disease, cardiotoxic drugs, hypertrophic cardiomyopathy, arrhythmogenic right ventricular cardiomyopathy, restrictive cardiomyopathy, or Chagas disease, were excluded. Verification of DCM and cardiac sarcoidosis diagnoses was achieved through a detailed review of electronic medical records.

### CMR imaging protocol

All subjects underwent CMR evaluation utilizing 1.5-Tesla scanners from Siemens Healthineers (MAGNETOM Avanto or Aera; Munich, Germany). Our established implantable cardioverter-defibrillator (ICD) CMR protocol was employed [15]. Gradient echo cine imaging was performed in long- and short-axis projections to assess ventricular volumes and function, using the following parameters: repetition time/echo time of 2.8 ms/1.4 ms, flip angle of 15°, 8 mm slice thickness with a 2 mm gap for short-axis slices, 192 × 180 matrix, 374 × 400 mm field of view, and 25 reconstructed cardiac phases per cardiac cycle. An electrocardiogram-gated, free-breathing, and motion-corrected phase-sensitive inversion-recovery gradient-echo turbo fast low-angle shot sequence was utilized to acquire LGE imaging of the LV. The imaging was performed using a short-axis stack of 2D images, acquired 15–20 min after the intravenous administration of 0.2 mmol/kg of gadolinium contrast (Dotarem (gadoterate meglumine), Guerbet; Princeton, NJ, USA), followed by a 20 ml saline flush. The imaging parameters included a repetition time/echo time of 14.7 ms/1.2 ms, a flip angle of 10°, an inversion time of 420 ms, a matrix size of 256 × 218 ~ 254, an average in-plane resolution of 1.4–1.6 × 1.4–1.6 mm, slice thicknesses of 4 mm or 6 mm, and 16 shot averages.

### Image analysis

Image analyses were conducted by an experienced reader with over 9 years of experience in CMR analysis, which were subsequently reviewed by a second observer with more than 15 years of expertise. A third reader with over two decades of experience in CMR analysis was consulted to mediate discrepancies when necessary. All image readers and analysts were blinded to clinical and outcome data.

Ventricular structural and functional parameters of CMR imaging were analyzed using the cvi42 software (Circle Cardiovascular Imaging; Calgary, Alberta, Canada). LGE images were analyzed utilizing ADAS 3D LV software (ADAS 3D Medical SL; Barcelona, Spain) [15]. In each short-axis image, the sub-endocardial and sub-epicardial borders of the LV were contoured semi-automatically. Tissue characterization on LGE images was performed by applying thresholds to distinguish between healthy myocardium (< 40% maximal signal intensity), border zone tissue (40–60% maximal signal intensity), and dense LGE regions or core (> 60% maximal signal intensity), with default thresholds set at 40% and 60%. Regions of the myocardium affected by ICD lead or generator artifact were manually excluded from the analysis during the process, ensuring that areas susceptible to device-related signal distortion do not influence our quantitative assessment of myocardial signal intensity [16].

**Fig. 1:**
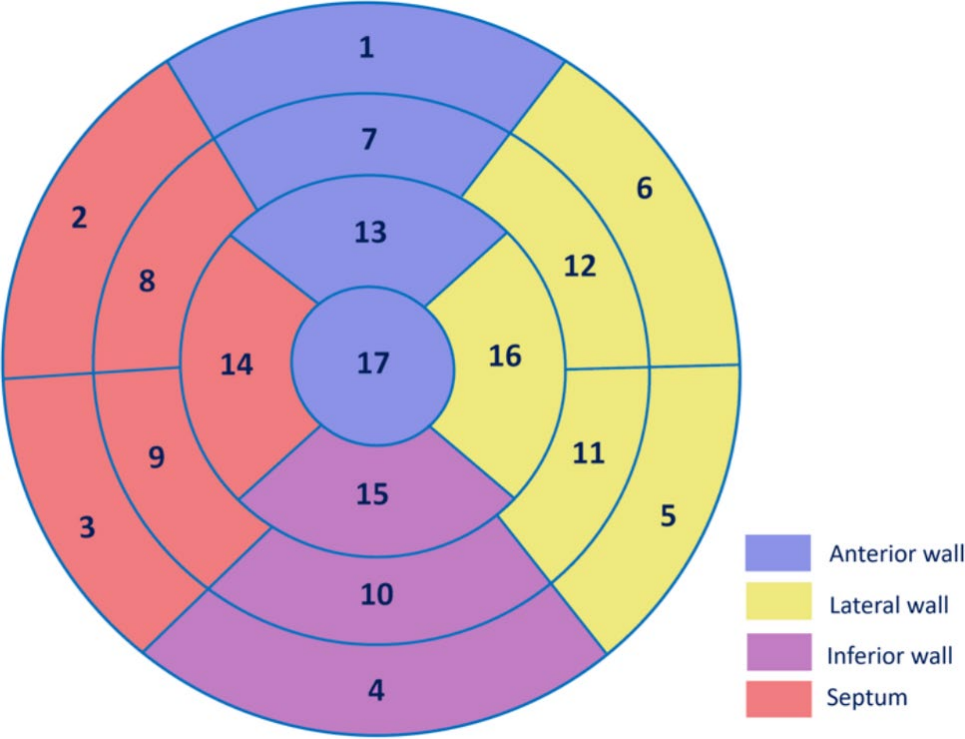
Illustration of the American Heart Association’s 17-segment model for myocardial segmentation with assignment of segment to various myocardial walls. The myocardial segmentation of the LV was divided based on American Heart Association (AHA) 17-segment model with segments 1, 7, 13, and 17 classified as the anterior wall; segments 5, 6, 11, 12, and 16 as the lateral wall; segments 4, 10, and 15 as the inferior wall; and segments 2, 3, 8, 9, and 14 as the septal wall

**Fig. 2:**
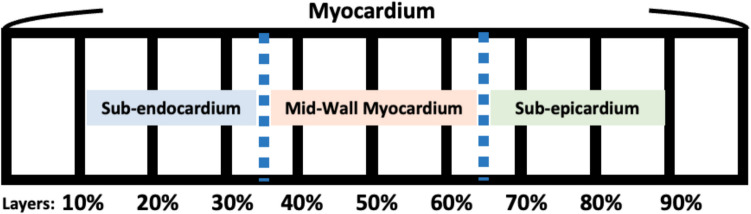
Demonstration of layered segmentation of the myocardium for cardiac magnetic resonance imaging analysis. The myocardium was equally spaced into nine layers, with each layer reflecting an increase from 10 to 90% of the total thickness from the sub-endocardium (10 to 30% of myocardial thickness), mid-wall (40 to 60% of myocardial thickness) to the sub-epicardium (70 to 90% of myocardial thickness)

The myocardial segmentation of the LV was further divided according to the American Heart Association (AHA) 17-segment model using the ADAS 3D LV software, with segments 1, 7, 13, and 17 classified as the anterior wall; segments 5, 6, 11, 12, and 16 as the lateral wall; segments 4, 10, and 15 as the inferior wall; and segments 2, 3, 8, 9, and 14 as the septal wall (Fig. 1) [17]. The myocardium was further divided into nine equally spaced layers, with each layer reflecting an increase from 10 to 90% of the total myocardial thickness. The layers were designated as sub-endocardial (10 to 30% of myocardial thickness), mid-wall (40 to 60% of myocardial thickness), and sub-epicardial (70 to 90% of myocardial thickness), as detailed in Fig. 2. Notably, in the interventricular septum—where the myocardium borders the RV cavity rather than the pericardial surface—the septal “sub-epicardial” layer is equivalent to the RV side sub-endocardium (or “RV endocardial third”).

CMR signal intensity, core, and border zone percentages were automatically computed for each myocardial segment and layer. The mean values for these parameters within each myocardial wall were obtained by averaging their measures across corresponding segments. Additionally, CMR signal intensity *z*-scores were computed using the formula *z* = (*x* – *μ*)/*σ*, where *x* represents the CMR signal intensity for a specific myocardial segment (according to the American Heart Association’s 17-segment model for the LV) of a specific layer (ranging from 10 to 90% of the myocardial thickness), and *μ* and *σ* denote the mean and standard deviation of CMR signal intensities across all myocardial segments and layers of the entire LV, respectively.

### Statistical analysis

**Fig. 3:**
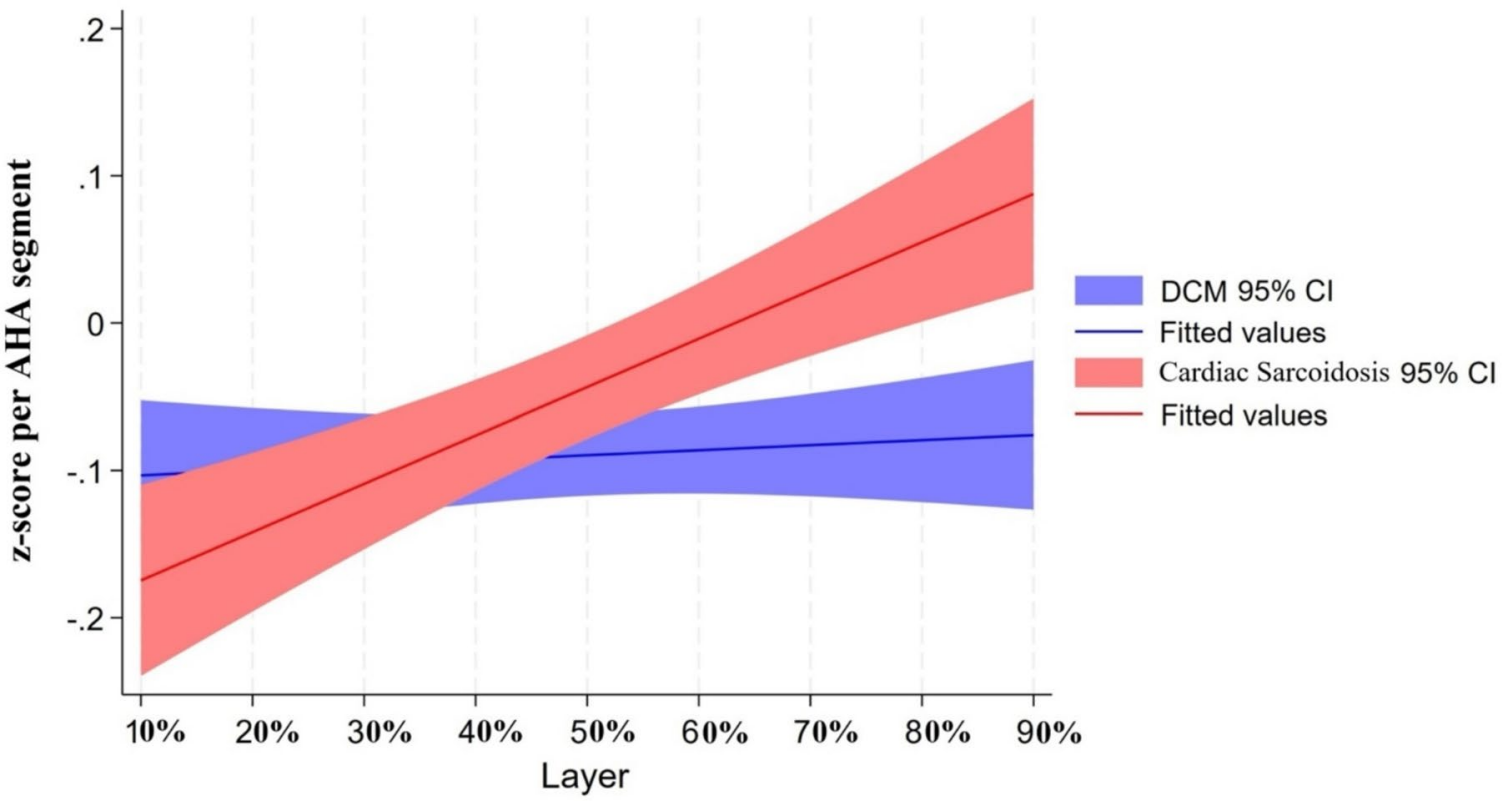
*z*-score variations between dilated cardiomyopathy and cardiac sarcoidosis in the septum: a locally weighted scatterplot smoothing curve analysis. The figure presents a locally weighted scatterplot smoothing (LOWESS) curve analysis of septal standardized CMR signal intensity *z*-scores on the *Y* axis and layer depth on the *X* axis, with the 10% layer closest to the left ventricular endocardium and the 90% layer closest to the right ventricular endocardium. Gadolinium uptake and hence CMR signal intensity *z*-scores were significantly higher in cardiac sarcoidosis near the right ventricular endocardium compared to dilated cardiomyopathy

**Fig. 4:**
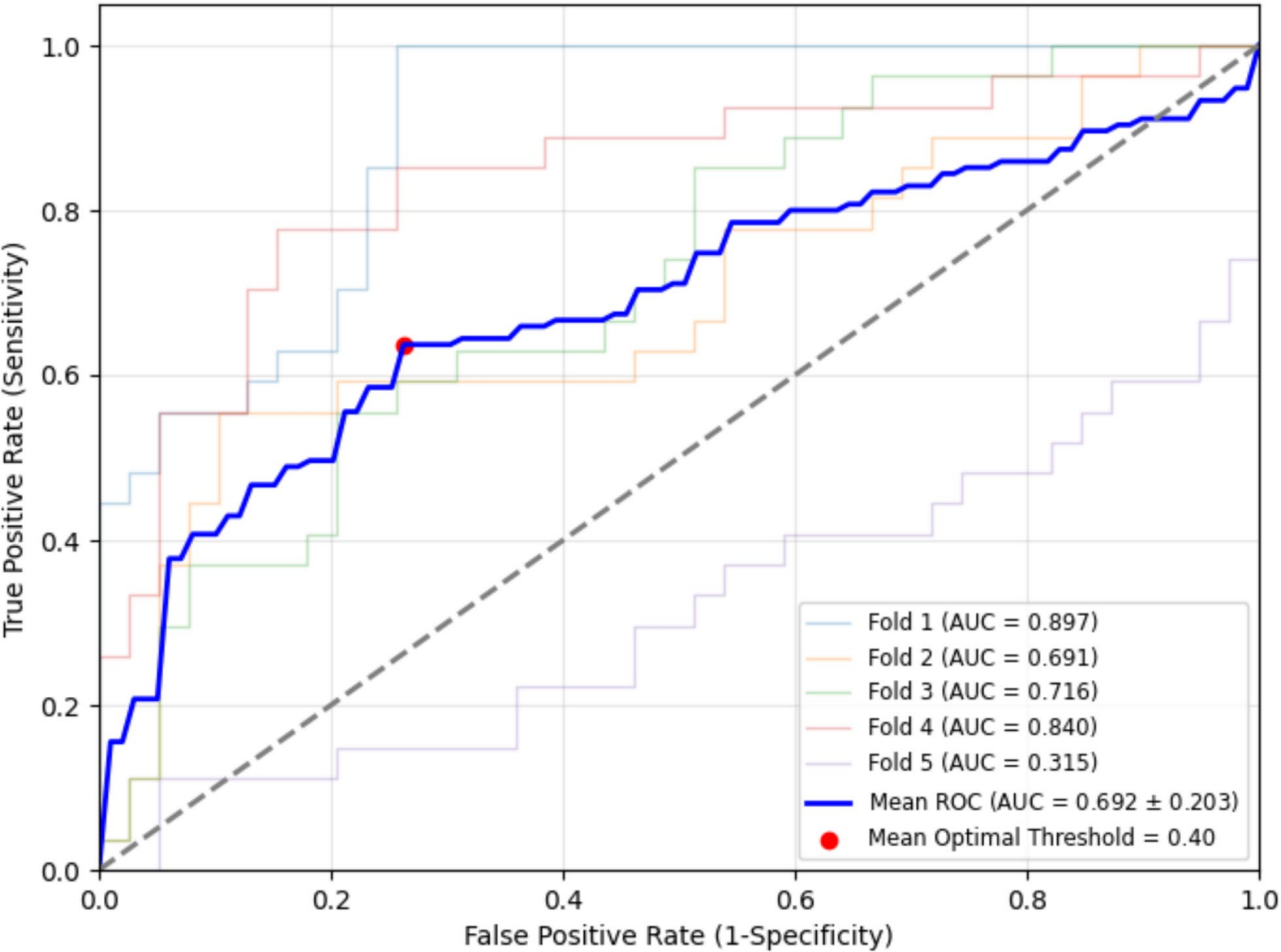
Receiver operating characteristic (ROC) curve for cardiac magnetic resonance signal intensity*z*-scores threshold in the endocardial third of the right ventricular septum (epicardial third of the left ventricular septum). The figure displays cross-validated receiver operating characteristic (ROC) curves as a graphical representation of the true positive rate (sensitivity) against the false positive rate (1 − specificity) for different thresholds of RV endocardial CMR signal intensity z -score for distinguishing between dilated cardiomyopathy and cardiac sarcoidosis. Each line represents one of the five folds in the fivefold cross-validation, whereby the dataset is randomly split into 5 subsets, and the model is trained and evaluated 5 times, each time using a different subset for evaluation and the rest for training resulting in AUC range from 0.315 to 0.897. The blue line denotes the mean ROC curve, aggregating the data across all folds, with a calculated mean AUC of 0.692 ± 0.203. The optimal diagnostic threshold, identified using Youden’s J statistic, is marked by a red dot at 0.40, optimizing the sensitivity and specificity for the diagnosis of cardiac sarcoidosis. At this threshold, the optimal mean sensitivity is 0.64 and the specificity is 0.74. Assuming the prevalence of cardiac sarcoidosis in NICM patients is similar to that in our cohort, approximately 40.9%, the positive predictive value (PPV) is 0.63 and the negative predictive value (NPV) is 0.75

The distribution of continuous variables was tested by the Shapiro–Wilk normality test. Continuous variables were presented as means ± standard deviation (SD) or median (25 th–75 th percentile) as appropriate. Categorical variables were described using frequencies and percentages. Continuous (or categorical) variables were compared between DCM and cardiac sarcoidosis groups using a *t*-test (or Mann–Whitney test) or chi-squared (or Fisher’s exact tests), as appropriate. Multi-level random effects logistic regression models, clustered by patient and by layers of the LV shell, were utilized to investigate the association between the etiology of cardiomyopathy (DCM vs. cardiac sarcoidosis) and variations in myocardial CMR signal intensity (*z*-score). These models were compared among walls (anterior wall, lateral wall, inferior wall, and septum, Fig. 1) and myocardial layers (sub-endocardium, mid-myocardium, and sub-epicardium, Fig. 2). The differences in CMR signal intensity *z*-scores between DCM and cardiac sarcoidosis in the septum were visualized using locally weighted scatterplot smoothing (LOWESS) curve analysis, as depicted in Fig. 3. For each patient, we determined the CMR signal intensity *z*-score value of the sector of the endocardial third of the RV septum. We then constructed an ROC curve (Fig. 4) using these *z*-score values from the specified region, with cardiac sarcoidosis as the binary outcome variable. We identified the threshold that maximized Youden’s index (sensitivity + specificity − 1). Finally, we employed a fivefold cross-validation method to validate the threshold’s performance on different subsets of the data.

Statistical analyses were performed using STATA (version 18.0; StataCorp LP, College Station, TX, USA) and Python (version 3.12; Python Software Foundation, Wilmington, DE, USA) running on Visual Studio Code (version 1.89.1). Data manipulation and analyses utilized Pandas (version 2.2.2), Scikit-learn (version 1.5.0) including StratifiedKFold, and Openpyxl (version 3.1.3). The receiver operating characteristic (ROC) curve was generated using Matplotlib (version 3.9.0). Statistical significance was established at a *p* value of less than 0.05 for all tests.

## Results

### Population demographics

The retrospective cohort study comprised 22 patients, including 13 patients with DCM (1 female) and 9 patients with cardiac sarcoidosis (1 female).

Of the 9 cardiac sarcoidosis patients:

- 2 were diagnosed by intramyocardial biopsy, meeting the histologic diagnosis group criteria for isolated cardiac sarcoidosis (JCS 2016).
- 5 were diagnosed by positive ^18^F-FDG PET findings plus three major criteria, fulfilling the clinical diagnosis group criteria for isolated cardiac sarcoidosis (JCS 2016).
- 2 had extracardiac biopsy-proven sarcoidosis who met the JCS 2016 criteria for systemic sarcoidosis with evidence of cardiac involvement on CMR and ^18^F-FDG PET.

**Fig. 5:**
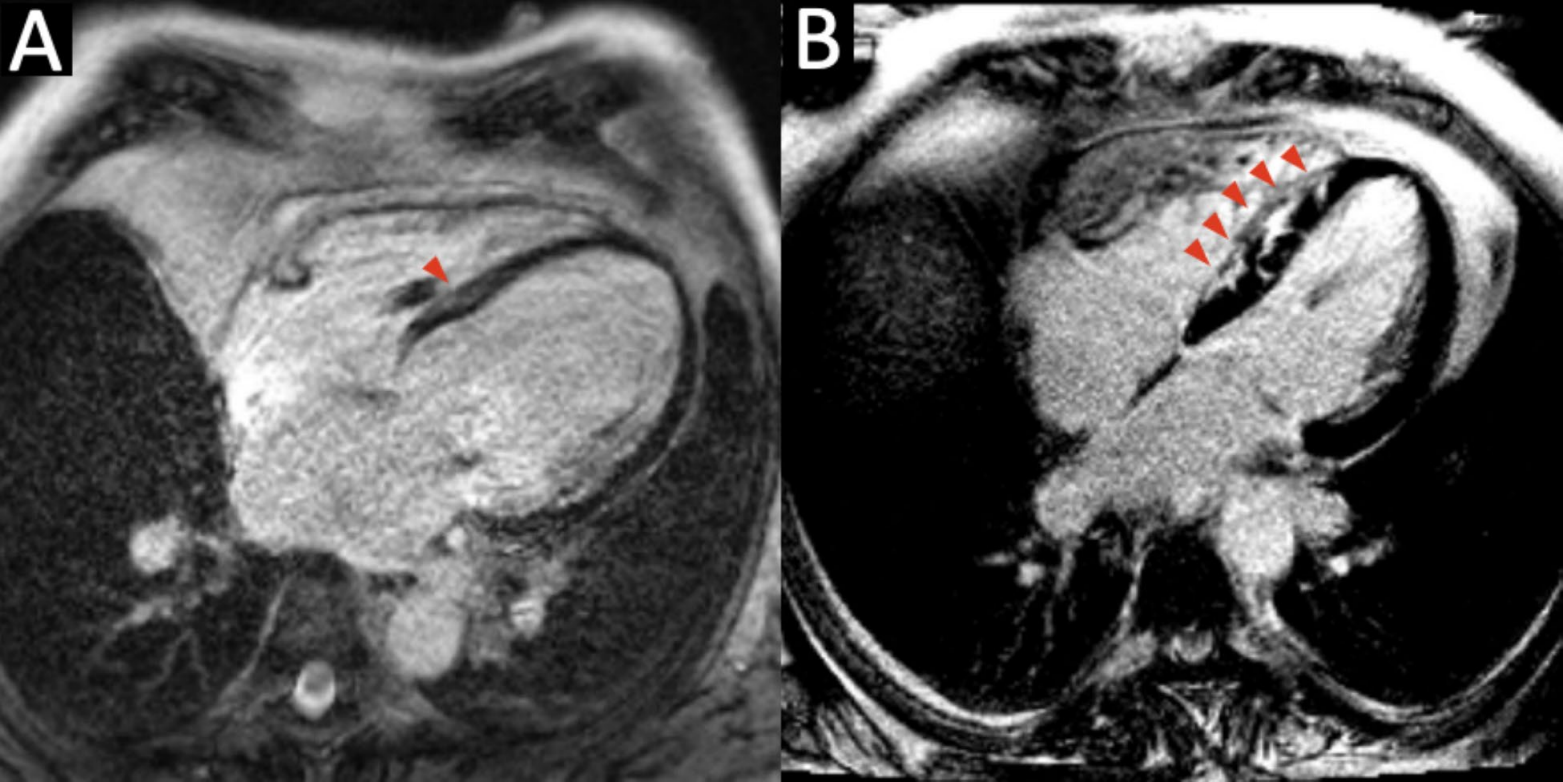
Demonstration of cardiac magnetic resonance imaging in dilated cardiomyopathy and cardiac sarcoidosis. Both panels present the four-chamber view of cardiac magnetic resonance imaging (CMR). **A** A patient with dilated cardiomyopathy (DCM), featuring mid-myocardial enhancement in the septum and a notably dilated left ventricle, indicated by the arrow. **B** A patient with cardiac sarcoidosis, highlighting multifocal late gadolinium enhancement in the interventricular septum, as indicated by the arrows

Compared to the cardiac sarcoidosis group, the DCM group tended to be older (mean 60.2 vs. 51.7 years, *p* = 0.016). Compared to the cardiac sarcoidosis group, the DCM group had significantly fewer patients on beta-blockers (30.8% vs. 88.9%, *p* = 0.01). Additionally, while the presence of ICD at the time of CMR was significantly higher in the DCM group compared to the cardiac sarcoidosis group (84.6% vs. 33.3%, *p* = 0.0447), four cardiac sarcoidosis patients received ICD implants immediately following electrophysiology study. Consequently, by the end of the day of CMR, 7 out of 9 (77.8%) cardiac sarcoidosis patients had ICDs. Baseline vital signs, cardiovascular disease history, risk factors, and other cardiovascular medication use did not differ significantly between the groups (Table 1). Figure 5 presents representative CMR images from one patient each with DCM (Fig. 5A) and cardiac sarcoidosis (Fig. 5B), illustrating typical imaging features observed in these conditions.

**Table 1:** Baseline characteristics of patients with dilated cardiomyopathy vs. cardiac sarcoidosis

Vital statistics	DCM (*n* = 13)	Sarcoidosis (*n* = 9)	*p* value
Age, year	60.2 ± 6.2	51.7 ± 9.0	0.016
Female	1 (7.8%)	1 (11.1%)	0.642
Body mass index, kg/m^2^	30.1 ± 7.3	31.5 ± 4.5	0.640
Disease history			
Heart failure	8 (61.5%)	6 (66.7%)	0.584
Hypertension	6 (46.2%)	6 (66.7%)	0.415
Diabetes mellitus	2 (15.4%)	3 (33.3%)	0.609
Other arrhythmia (Afib/AFL or PVC)	8 (61.5%)	3 (33.3%)	0.387
Pulmonary disease	2 (15.4%)	1 (11.1%)	0.642
Renal insufficiency	2 (15.4%)	1 (11.1%)	0.642
Hyperlipidemia	2 (15.4%)	0 (0.0%)	0.308
Hypothyroidism	2 (15.4%)	2 (22.2%)	0.550
Obstructive sleep apnea	2 (15.4%)	1 (11.1%)	0.642
Presence of ICD*	11 (84.6%)	3 (33.3%)	0.045
Laboratory test			
Serum creatinine, mg/dl	1.2 ± 0.3	1.2 ± 0.3	0.518
Antiarrhythmic medications			
Amiodarone	5 (38.5%)	4 (44.4%)	0.561
Sotalol	3 (23.1%)	0 (0.0%)	0.240
Other medications			
Antiplatelet medications	4 (30.8%)	2 (22.2%)	0.523
Anticoagulation medications	6 (46.2%)	3 (33.3%)	0.674
Statin	9 (69.2%)	3 (33.3%)	0.192
ACE inhibitor or ARB use	9 (69.2%)	4 (44.4%)	0.384
Beta-blocker use	4 (30.8%)	8 (88.9%)	0.011
Mineralocorticoid receptor antagonist	3 (23.1%)	3 (33.3%)	0.655

^*^ICD status was recorded at the time of CMR. Four cardiac sarcoidosis patients and one DCM patient subsequently underwent ICD implantation following CMR, while the remaining patients did not meet criteria for device placement (i.e., insufficient duration on guideline-directed medical therapy or an LVEF > 35%)

Continuous variables are expressed as means ± SD or medians (25 th to 75 th percentile), as appropriate, and categorical variables are expressed as numbers (percentages). A *p* value of less than 0.05 was considered to indicate statistical significance for all tests. Abbreviations: *Afib/AFL* atrial fibrillation/flutter, *CMR* cardiovascular magnetic resonance imaging, *PVC* premature ventricular contraction, *ICD* implantable cardioverter-defibrillators, *ACEI* angiotensin converting enzyme inhibitor, *ARB* angiotensin II receptor blocker

### Cardiac structural and functional parameters from CMR

Patients with DCM exhibited enlarged LV end-systolic chamber (mean LVESVi 79.4 vs. 54.4 ml/m^2^; *p* = 0.025), compared to the cardiac sarcoidosis group. Further evaluations encompassing the LV function, LV end-diastolic chamber size, LV mass, LV wall thicknesses, RV function and volume, left and right atrial dimension, and ascending aorta diameter did not reveal significant differences between the two groups (Table 2).

**Table 2:** Comparative analysis of cardiac magnetic resonance imaging structural and functional parameters in patients with dilated cardiomyopathy vs. cardiac sarcoidosis

	DCM (*n* = 13)	Sarcoidosis (*n* = 9)	*p* value
LVEF, %	35.4 ± 10.8	41.5 ± 9.1	0.1787
LVEDVi, ml/m^2^	119.7 ± 36.7	96.6 ± 13.9	0.0878
LVESVi, ml/m^2^	79.4 ± 29.4	54.4 ± 11.4	0.0254
LV Massi, g/m^2^	65.4 ± 15.7	65.4 ± 15.7	0.5307
LV anteroseptal wall thickness (mm)	11.6 ± 4.3	10.6 ± 2.8	0.5896
LV inferolateral wall thickness (mm)	8.0 ± 2.9	9.1 ± 2.7	0.4535
RVEF, %	39.8 ± 8.3	40.1 ± 8.2	0.9517
RVEDVi, ml/m^2^	91.0 ± 14.0	106.2 ± 21.9	0.0595
RVESVi, ml/m^2^	55.1 ± 13.3	63.5 ± 22.4	0.2801
LA 4 chamber length (mm)	59.9 ± 11.4	54.0 ± 5.4	0.1984
RA 4 chamber length (mm)	53.4 ± 9.3	57.5 ± 9.3	0.3642
Ascending aorta size (mm)	29.8 ± 10.0	33.4 ± 4.6	0.365

Continuous variables are expressed as means ± SD or medians (25 th to 75 th percentile), as appropriate. A *p* value of less than 0.05 was considered to indicate statistical significance for all tests. Abbreviations: *DCM* dilated cardiomyopathy, *LA* left atrium, *LVEDV* left ventricular end-diastolic volume, *LVEDVi* left ventricular end-diastolic volume indexed, *LVEF%* left ventricular ejection fraction, *LVESV* left ventricular end-systolic volume, *RA* right atrium, *RVEDV* right ventricular end-diastolic volume, *RVESV* right ventricular end-systolic volume, *RVEF%* right ventricular ejection fraction

### Septal LGE involvement

Septal LGE was observed more frequently in patients with cardiac sarcoidosis compared to those with DCM. In the cardiac sarcoidosis cohort (*n* = 9), septal LGE was present in 7 patients, with 4 exhibiting a patchy transmural pattern, 1 patient showing enhancement confined to the RV sub-endocardial layer of the septum (equivalent to the “sub-epicardial” layer in our segmental analysis), and 2 demonstrating a subepicardial-to-mid pattern. With respect to the basal-to-apical distribution, the distribution extended from the basal to apical segments in 2 cases, from the basal to mid segments in 3 cases, and was confined exclusively to the basal or apical segments in 1 case each. In contrast, among patients with DCM (*n* = 13), septal LGE was identified in only 4 patients, all of whom exhibited either mid-wall (*n* = 2) or subepicardial (*n* = 2) enhancement, confined to the basal septum.

### Border zone, core, and combined border zone + core involvement

Table 3 demonstrates a detailed regression analysis examining the degree of myocardial involvement in patients with DCM vs. those with cardiac sarcoidosis. The analysis quantifies border zone involvement, core involvement, and combined border zone and core involvement across different myocardial wall locations (anterior, lateral, inferior walls, and septum). Regression coefficients are provided to compare the extent of cardiac sarcoidosis involvement against DCM, serving as the reference group.

**Table 3:** Regression analysis percentage of border zone, core, and combined involvement in dilated cardiomyopathy vs. cardiac sarcoidosis

	Location of myocardium wall	*β* (sarcoid vs. DCM (reference))	*p* value
BZ + core%	Anterior wall n* = 776	− 11.41 (− 40.80, 17.99)	0.447
	Lateral wall n* = 990	− 9.62 (− 38.03, 18.79)	0.507
	Inferior wall n* = 594	− 21.46 (− 52.05, 9.14)	0.169
	Septum *n* *= 990	− 10.89 (− 42.54, 20.77)	0.5
core%	Anterior wall n*= 776	− 0.08 (− 12.01, 11.85)	0.989
	Lateral wall n* = 990	− 4.82 (− 13.71, 4.06)	0.287
	Inferior wall *n* *= 594	− 7.46 (− 21.76, 6.85)	0.307
	Septum n* = 990	5.05 (− 5.64, 15.73)	0.355
BZ%	Anterior wall n*= 776	− 11.32 (− 34.82, 12.19)	0.345
	Lateral wall n* = 990	− 4.80 (− 28.55, 18.95)	0.692
	Inferior wall n* = 594	− 14.00 (− 37.95, 9.95)	0.252
	Septum n* = 990	− 15.93 (− 42.38, 10.52)	0.238

Continuous variables are expressed as means ± SD or medians (25 th to 75 th percentile), as appropriate. *Number of AHA segments available; a *p* value of less than 0.05 was considered to indicate statistical significance for all tests

Across the measured parameters, no statistically significant differences were observed between the two patient groups. Notably, both the percentages of border zone involvement and combined border zone and core involvement frequently exhibited negative coefficients when comparing cardiac sarcoidosis to DCM. In contrast, the percentage of core involvement in the septum exhibited a positive trend (mean coefficient *β* = 5.05%; 95% CI, − 5.64 to 15.73%) relative to DCM, which, did not reach statistical significance (*p* = 0.345).

### *z*-score analysis of CMR signal intensities across myocardial locations and layers

Table 4 details the regression analysis of CMR signal intensity *z*-scores across various myocardial wall locations (anterior wall, lateral wall, inferior wall, and septum) and layers (sub-endocardium, mid-myocardium, and sub-epicardium) in patients diagnosed with DCM vs. those with cardiac sarcoidosis. The analysis reveals that *z*-scores are significantly higher in the septum of patients with cardiac sarcoidosis compared to those with DCM, with a positive regression coefficient of 0.32 (95% CI, 0.08, 0.56; *p* = 0.009).

**Table 4:** Regression analysis of*z*-score comparisons by myocardial wall and layer-specific in patients with dilated cardiomyopathy vs. cardiac sarcoidosis

Location of myocardium wall	*β* (sarcoid vs. DCM (reference))	*p* value
Anterior wall Number of AHA segment = 776	0.13 (− 0.15, 0.42)	0.354
Lateral wall *n* = 990	− 0.16 (− 0.41, 0.09)	0.223
Inferior wall *n* = 594	− 0.19 (− 0.49, 0.10)	0.198
Septum *n* = 990	**0.32 (0.08, 0.56)**	**0.009**
Anterior wall		
Sub-endo *n* = 255	0.12 (− 0.13, 0.37)	0.351
Mid-ventricle *n* = 260	0.08 (− 0.22, 0.38)	0.614
Sub-epi *n* = 261	0.22 (− 0.13, 0.56)	0.216
Lateral wall		
Sub-endo *n* = 330	− 0.16 (− 0.42, 0.09)	0.209
Mid-ventricle *n* = 330	− 0.22 (− 0.48, 0.05)	0.106
Sub-epi *n* = 330	− 0.09 (− 0.40, 0.23)	0.589
Inferior wall		
Sub-endo *n* = 198	− 0.20 (− 0.49, 0.08)	0.155
Mid-ventricle *n* = 198	− 0.26 (− 0.57, 0.06)	0.108
Sub-epi *n* = 198	− 0.12 (− 0.46, 0.22)	0.5
Septum		
LV Sub-endo *n* = 330	0.14 (− 0.09, 0.37)	0.222
Mid-ventricle *n* = 330	0.27 (− 0.01, 0.55)	0.06
RV sub-endo (LV sub-epi) *n* = 330	**0.56 (0.22, 0.89)**	**0.001**

Continuous variables are expressed as means ± SD or medians (25 th to 75 th percentile), as appropriate. *Number of AHA segment available; a *p* value of less than 0.05 was considered to indicate statistical significance for all tests

Data in bold emphasis indicates statistical significance

Moreover, the myocardial layer-specific analysis largely aligned with the findings for wall locations, indicating similar trends where cardiac sarcoidosis exhibited higher *z*-scores than DCM in the anterior wall and septum, and DCM demonstrated higher *z*-scores in the lateral and inferior walls. However, these differences were statistically significant only in the RV endocardial side of the septum, where *z*-scores for cardiac sarcoidosis significantly exceeded those for DCM (*β* [cardiac sarcoidosis vs. DCM (reference)] = 0.56; 95% CI, 0.22, 0.89; *p* = 0.001). The statistical details are comprehensively provided in Table 4. Furthermore, Fig. 3 provides a graphical representation of the *z*-score variations between DCM and cardiac sarcoidosis in the septum, using a LOWESS curve analysis to visually highlight the differential CMR signal intensities across myocardial layers.

The ROC curve, visualized in Fig. 4 and subjected to fivefold cross-validation, demonstrates the diagnostic performance of septal CMR signal intensity *z*-scores. We identified an optimal threshold of approximately 0.40 in the RV endocardial third of the septum, achieving an optimal mean sensitivity of 0.64 and a specificity of 0.74. The average AUC was 0.692 ± 0.203 across the folds. Assuming the prevalence of cardiac sarcoidosis in NICM patients is similar to that in our study population (9 out of 22 cases, approximately 40.9%), we calculated a positive predictive value (PPV) of 0.63 and a negative predictive value (NPV) of 0.75 for the septal RV endocardial z-score threshold set at 0.40.

## Discussion

In this study, we analyzed CMR imaging signal intensities using the *z*-score methodology to elucidate the patterns and textures of myocardial scar involvement in DCM and cardiac sarcoidosis. The main findings are as follows:

1. The septum displayed higher signal intensity *z*-scores in cardiac sarcoidosis than in DCM. This trend persisted across the myocardial layers (sub-endocardial, mid-myocardial, and sub-epicardial) in the LV septum, although statistical significance was noted exclusively within the endocardial third of the RV septum.
2. A threshold signal intensity *z*-score of 0.40 in the endocardial third of the RV septum was associated with a diagnosis of cardiac sarcoidosis, with an AUC of 0.692 on fivefold cross-validation.

Our observations align with previous research delineating distinct LGE patterns/textures in DCM and cardiac sarcoidosis. A comprehensive analysis of the DCM Precision Medicine Study revealed that LGE in DCM primarily manifests in mid-myocardial or epicardial regions of the LV [18]. In contrast, a meta-analysis focusing on histologically confirmed cases of cardiac sarcoidosis uncovered a significant pattern of sub-epicardial, multifocal, and septal involvement in the LV [19]. Multiple investigations have demonstrated that LGE in cardiac sarcoidosis predominantly affects the septum, exhibiting a multifocal and patchy distribution, with the sub-epicardial layers or RV side of the ventricular septum commonly involved, although all layers may be involved [9, 20, 21]. This discrepancy between DCM and cardiac sarcoidosis in LGE manifestation aligns with our findings from the *z*-score analysis, where cardiac sarcoidosis demonstrated significantly higher signal intensity *z*-scores in the epicardial LV septum, i.e., the endocardial RV septum when compared to DCM. In our experience, this predominantly basal RV endocardial septal distribution of scar is closely associated with the incidence of right bundle branch block and conduction system disease, as well as critical VT circuitry components in cardiac sarcoidosis. The discernible patterns/textures of myocardial involvement, as identified through the *z*-score methodology applied to CMR signal intensities between patients with DCM and cardiac sarcoidosis, underscore the analytical value of *z*-score in the objective quantification of myocardial intensity variations among patients. Additionally, the observed discrepancies in the core percentage—defined as the dense LGE regions or core (> 60% maximal signal intensity)—suggesting a predominance in cardiac sarcoidosis over DCM within the septum, albeit not statistically significant, may offer further evidence towards the heightened signal intensity *z*-scores in the septal region.

LGE has been established as a pivotal diagnostic tool in the context of NICM, attributed to its proficiency in identifying macroscopic myocardial scars [10, 22]. The extent, topography, and pattern of LGE are powerful correlates associated with worse outcomes in patients with NICM, serving as critical markers for identifying individuals at an elevated risk for adverse events. Consequently, LGE has been incorporated into current cardiomyopathy management guidelines [1, 22, 23]. Nonetheless, the inherently subjective nature of LGE assessment, compounded by the paucity of quantitative analytical techniques on standard-of-care CMR, has prompted the exploration of novel methodologies to circumvent these limitations. Drawing inspiration from prior research focused on the intensity standardization of CMR signals across various anatomical structures, including the brain and head and neck cancers, the present study adopts the *z*-score method [11, 12]. While LGE imaging predominantly enables the identification of macroscopic myocardial scarring, the *z*-score methodology, by virtue of its ability to detect elevated signal intensities within various regions of interest, may objectively unveil microscopic pathological alterations that remain occult on LGE imaging along with the demonstration of macroscopic myocardial scarring distribution. However, further clinical investigations are imperative to substantiate the correlation between histological findings, electroanatomic mapping, and the *z*-score approach.

In CMR, a range of magnetic resonance imaging (MRI) techniques, including T1 and T2 mapping as well as quantitative perfusion CMR, have been employed for the quantitative assessment of cardiac morphology, tissue properties, and function [24]. T1 mapping, in particular, has emerged as a pivotal tool for delineating myocardial fibrosis, offering prognostic value by correlating with disease severity, including various studies in DCM [25, 26]. This is exemplified in studies involving patients with NICM without LGE, where diffuse fibrosis identified through post-contrast T1 mapping has been linked to electrical abnormalities on electroanatomic mapping [27]. T2 mapping plays an essential role in identifying myocardial edema, indicative of acute inflammation or injury, thus crucial for differentiating acute and chronic cardiac conditions [24]. For example, Crouser et al. have shown that myocardial T2 mapping, when used in conjunction with LGE, significantly enhances the diagnostic efficacy for cardiac sarcoidosis, with elevated T2 values offering improved predictive insights into electrocardiographic abnormalities and arrhythmias beyond the capacity of either technique alone [28]. Despite these advancements, the broader application of these quantitative methods is often limited by the need for additional imaging acquisitions and the availability of such sophisticated analyses across all centers. In response, our methodology, which integrates *z*-score analysis derived from routine standard-of-care MRI scans, offers an efficient alternative for quantitative evaluation without the need for supplementary imaging acquisitions.

The *z*-score methodology for CMR signal intensities provides an objective and quantitatively measurable way to evaluate myocardial scar involvement in patients with NICM. This method enables consistent comparisons across different CMR scans, enhancing the clinical value of standard-of-care CMR protocols. It also sets the stage for the incorporation of standardized, quantifiable CMR metrics into cardiomyopathy clinical guidelines. Furthermore, by integrating the *z*-score approach with insights from LGE, T1/T2 mapping, and new imaging technologies, we anticipate an improvement in the accuracy of diagnosing NICM scar involvement. This progress could lead to earlier and more precise identification of myocardial conditions in NICM patients through CMR imaging, improving patient outcomes by enabling tailored treatment plans and proactive disease management.

## Limitations

The limitations of our study include a relatively small sample size and its retrospective nature. Future studies will be needed to prospectively validate these results with a larger sample size and to enhance generalizability. Not all patients with DCM underwent genetic testing; therefore, some substrate heterogeneity due to the presence of LMNA and other genetic myopathies may exist in the DCM cohort. The *z*-score methodology provides a standardized approach but is a novel tool in the realm of CMR imaging for patients with NICM. Furthermore, a significant proportion of the study participants, 14 out of 22 (63.6%), had ICDs at the time of CMR imaging. The prevalence of ICDs was notably higher in the DCM cohort, with 11 out of 13 patients (84.6%), compared to 3 out of 9 patients (33.3%) in the cardiac sarcoidosis group. Future prospective studies employing standardized imaging protocols and larger cohorts, as well as confirmatory studies correlating imaging findings with histological data, are crucial to validate and extend our findings.

## Conclusion

In this study, we employed the *z*-score methodology to calculate CMR signal intensities in myocardial regions of interest, facilitating quantitative comparisons of myocardial tissue characteristics between DCM and cardiac sarcoidosis. We found that *z*-score analysis offers a new objective perspective on interpreting LGE scars, with significant differences observed in *z*-score intensities, particularly in the septum and on the RV endocardial side. This approach could pave the way for future research to further validate and expand the clinical utility of standard-of-care MRI.

## Data Availability

Data supporting the findings of this study are available from the corresponding author, Dr. Saman Nazarian, upon reasonable request.
